# Mini-Open Latarjet Procedure for Recurrent Anterior Shoulder Instability

**DOI:** 10.4061/2011/656205

**Published:** 2011-12-05

**Authors:** Numa Mercier, Dominique Saragaglia

**Affiliations:** Department of Orthopaedic Surgery and Sport Traumatology, Grenoble South Teaching Hospital, 38130 Échirolles, France

## Abstract

Anterior shoulder instability is a common problem. The Latarjet procedure has been advocated as an option for the treatment of anteroinferior shoulder instability. The purpose of this paper is to explain our surgical procedure titled “Mini-open Latarjet Procedure.” We detailed patient positioning, skin incision, subscapularis approach, and coracoid fixation. Then, we reviewed the literature to evaluate the clinical outcomes of this procedure.

## 1. History of Coracoid Transposition

More than 150 operations have been described for the treatment of recurrent anterior dislocation of the shoulder [1]. The ideal surgical treatment renders the shoulder stable without compromising strength or range of motion. Transfer of the coracoid process through the subscapularis tendon is one of them.

### 1.1. Latarjet Procedure

This procedure was first described in 1954 by Latarjet [2] for the treatment of recurrent dislocation of the shoulder. The essential feature of this procedure was the transplantation of the coracoid process to the neck of the scapula through the subscapularis tendon. The coracoid process flat was laid with its posterior surface against the neck of the glenoid. The author used a screw to secure fixation of the coracoid to scapular neck.

### 1.2. Bristow Procedure

Helfet in 1958 [3], described the Bristow procedure in which the coracoid process was merely sutured to the anterior part of the scapular neck through a transversally sectioned subscapularis muscle. The object of this operation was to transplant the terminal half-inch of the coracoid, which carries the conjoined tendons to the neck of the scapula, just medial to the anterior-inferior edge of the glenoid rim. Only the cancellous end of the coracoid was fixed on the neck of the glenoid. Helfet, who credited this procedure to his former chief, Dr W. Rowley Bristow, later admitted that the coracoid transfer, in fact, was his own innovation, but he wanted to honor his former chief, who died 10 years prior to Helfet's procedure.

Mead and Sweeney in 1964 [4], and May in 1970 [5], described a modification of the Bristow Helfet procedure that consisted of fixing the bone block to the anterior glenoid rim with a screw.

### 1.3. Bristow-Latarjet Procedure

Coracoid transposition has been modified extensively. But modifications still usually involve transfer of the distal tip of the coracoid process with the attached conjoined tendon to the anterior rim of the glenoid through a split or division of the subscapularis muscle-tendon unit.

In the English literature, this procedure has become now as the Bristow-Latarjet operation.

## 2. Mini-Open Latarjet Procedure

### 2.1. Positioning

The patient is placed supine on the operating table in lying position in our practice and not in a beach chair position. A small roll can be positioned under the scapula of the involved side. The shoulder and upper extremity are draped free for some surgeons and only the shoulder for others.

### 2.2. Incision

At the beginning, to perform the Latarjet procedure [2], a standard deltopectoral approach was used. The incision begun one centimeter proximal to the coracoid process and was extended eight centimeters distally toward the anterior axillary fold [6].

**Figure 1:**
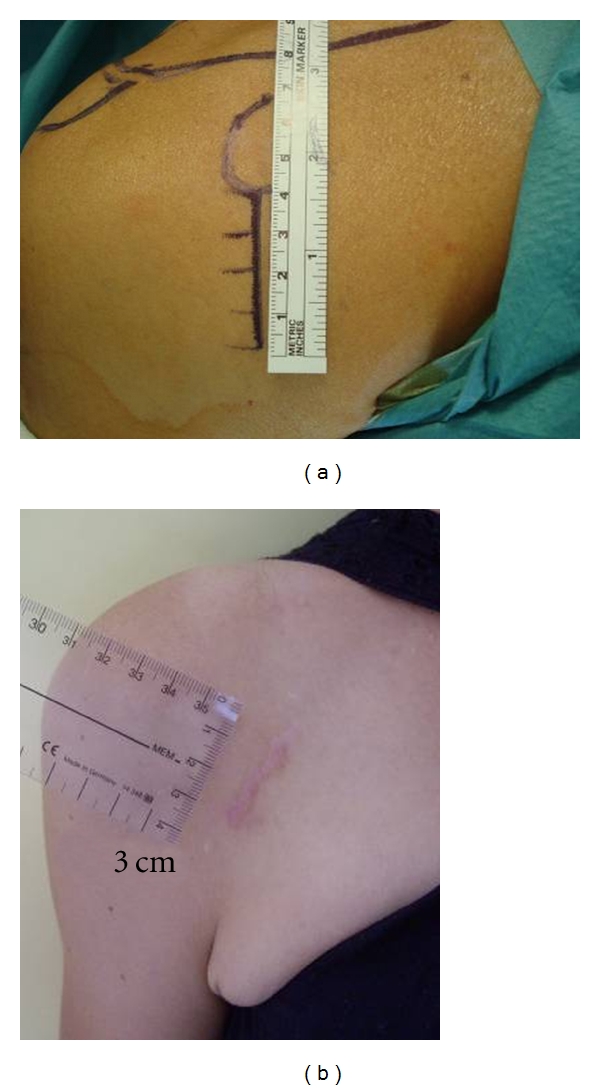
(a) Peroperatoire view of skin incision. (b) A small scare (3 cm) in a young woman.

Now a limited deltopectoral approach is used. The skin incision begins from the tip of the coracoid extending 4 cm toward the axillary fold. For fatty patients, the skin incision is longer than 4 cm but does not exceed 6 cm. When it is possible, a small incision, like 3 centimeters (Figure 1), is realized when patients are thin. This short incision is made possible by the skin elasticity at this site. Usually the subcutaneous dissection is more extensive than the skin incision.

### 2.3. Surgical Approach

**Figure 2:**
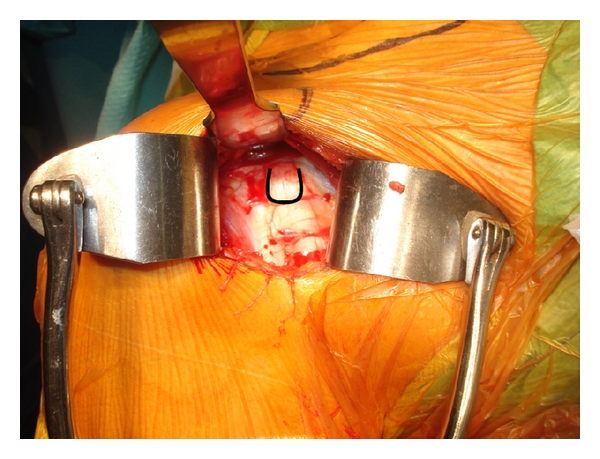
Exposure of the coracoid process on a right shoulder with a 4 cm skin incision.

The cephalic vein is protected and retracted laterally. The anterior deltoid is splitted up in order to reach the coracoid process and the conjoined tendon. Then a self-retaining retractor is inserted into the wound. The coracoid process is exposed from its tip to the insertion of the coracoclavicular ligaments at the base of the coracoid (Figure 2). The coracoacromial ligament is sharply dissected from the lateral aspect of the coracoid, and the pectoralis minor tendon insertion on the medial side of the coracoid is visualized.

### 2.4. Coracoid Preparation

The pectoralis minor tendon insertion is released with the electrocautery from the coracoid process as well as the coracoacromial ligament which is taken off at the level of its bony insertion. Then, a 4.5 mm diameter hole is drilled into the middle of the coracoid perpendicular to its upper side. The hole is threaded with a 6.5 mm cancellous screw tap before cutting the coracoid. Finally, a Pauwells osteotome is used to perform the osteotomy of the coracoid at the coracoid knee. The bone block measure 2 to 3 cm long. The bone block is turned over to remove the periosteum and to smooth over its shape. Another self-retaining retractor is inserted perpendicular to the first one in order to recline distally the coracoid.

### 2.5. Subscapularis Approach

Once the osteotomy of the coracoid has been performed, there is a clear view of the subscapularis tendon. The upper and inferior parts of the muscle are identified and 2 sutures are placed at its muscle and tendon junction to pull up the tendon in order to facilitate its incision.

**Figure 3:**
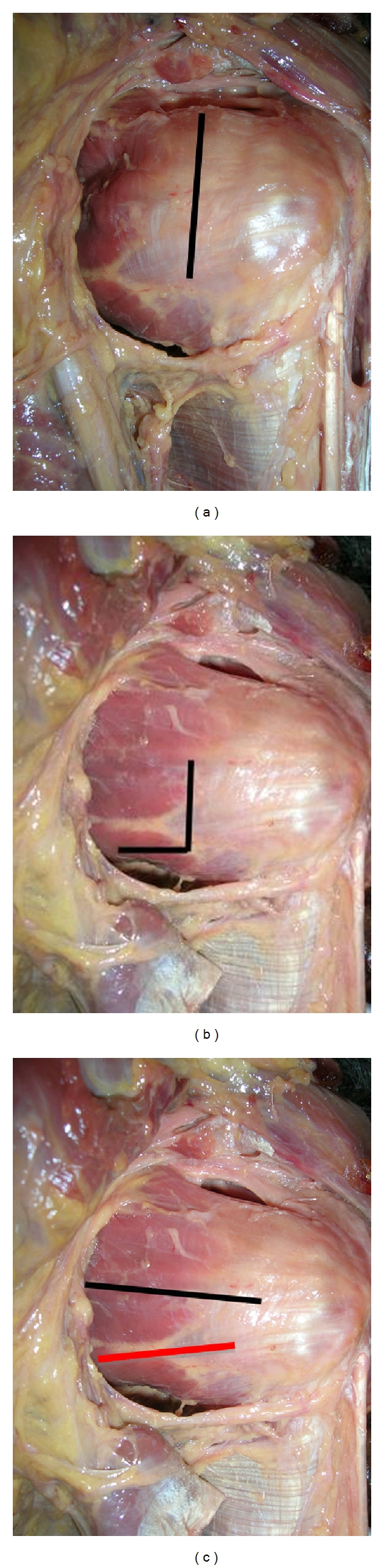
Incision technique for the subscapularis tendon (cadaver views). (a) Complete vertical section of the subscapularis. (b) Weaver incision of the subscapularis. (c) Horizontal incision at the lower end of the subscapularis (red line).

**Figure 4:**
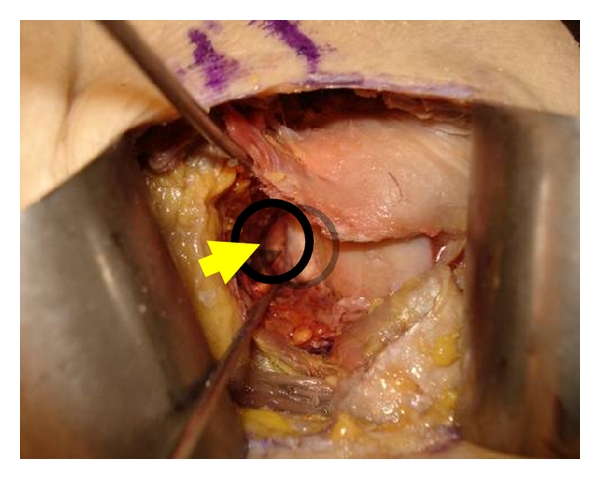
Exposure of the antero-inferior part of the glenoid (arrow) (cadaver view).

The incision technique for the subscapularis muscle tendon was modified during his surgical practice by the senior author (D. Saragaglia). Between 1981 and 1996 the senior author used a complete vertical section of the subscapularis [7]. Between 1996 and 2008, the way used was the Weaver section of the subscapularis [8], that is, to say a partial section of the lower third of the muscle preserving the upper part of the tendon (Figure 3). Now, we do not use any section but we split horizontally the subscapularis tendon at its lower part and the tendon is retracted in the upper part. A 2.2 mm diameter K-wire is hammered into the scapular neck as high as possible to maintain the retraction of the subscapularis tendon and a Hohmann retractor (or another K-wire) is placed into the lower part of the glenoid neck in order to improve the exposure of the neck of the scapula (Figure 4).

### 2.6. Glenoid Preparation

The capsule incision is performed with the electrocautery at the same time of the splitting of the inferior border of the subscapularis. Then, the anterior-inferior glenoid neck is prepared with an osteotome to decorticate the anterior surface.

### 2.7. Coracoid Positioning

Proper positioning of the coracoid bone graft relative to the glenoid is critical. Care is taken not to place the graft too far laterally or medially. It is not intended to be a bone block, and therefore it is placed so that it functions as an extension of the glenoid articular arc. A 3.2 mm hole is drilled, parallel to the joint line, one centimeter above the distal border of the glenoid rim and 0.5 cm medially to the glenoid cartilage. Then the hole is threaded with a 6.5 mm cancellous screw tap.

### 2.8. Coracoid Fixation

**Figure 5:**
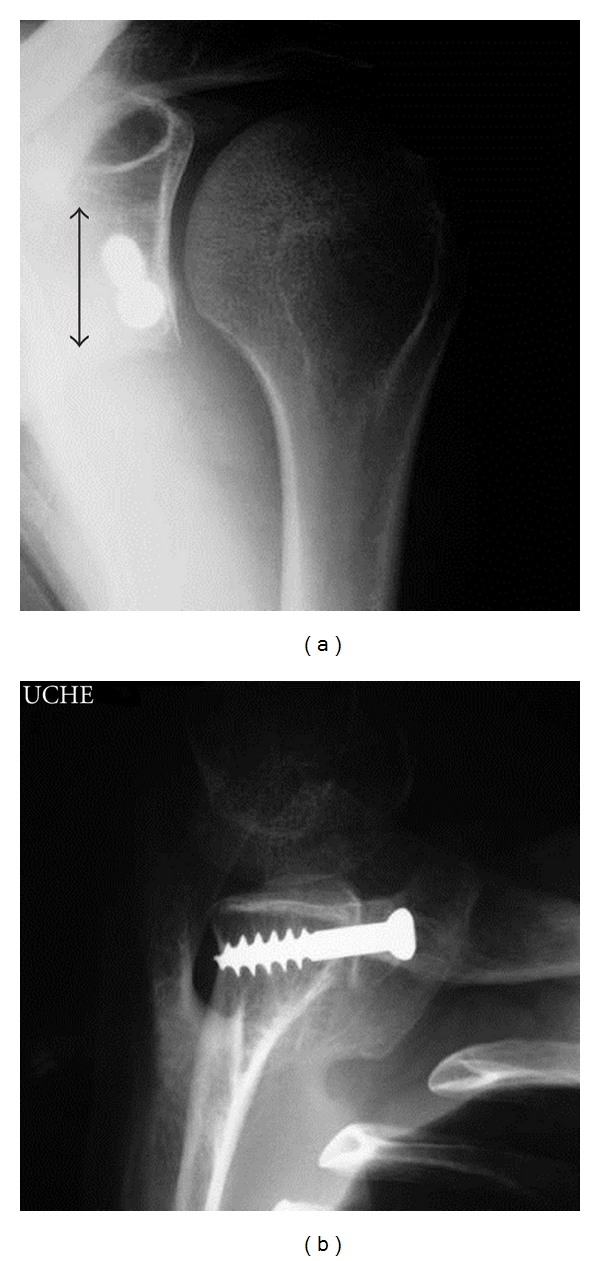
Good positioning of the bone block on AP (a) and Bernageau (b) views. The size of the graft is shown by the lenght of the arrow (a) (2 to 3 cm long).

A 35 mm length AO 6.5 mm cancellous screw is first screwed into the bone block without any washer, then the bone block is pushed down with the screw driver in order to put the tip of the screw in the glenoid hole, and finally the graft is screwed in right position (Figure 5).

Then the subscapularis tendon is shut on the conjoined tendon. A stitch is done between the subscapularis and the conjoined tendon to close the interval. Finally, a standard closure is performed.

### 2.9. Postoperative Care

Patients use a sling for 7 to 15 days only to reduce pain. Rehabilitation with mobilization in elevation and external rotation is allowed the day after surgery. Strengthening exercises on the biceps are delayed until 3 months postoperatively to protect the coracoid healing. At this time the bone graft usually shows early radiographic evidence of consolidation with the glenoid. Contact sports and heavy labor are generally allowed at 3 months postoperatively.

## 3. Clinical Outcomes of Bristow-Latarjet Procedure

### 3.1. Range of Motion

Loss of motion, especially external rotation, has long been a criticism of the modified Bristow-Latarjet procedure. Most authors have reported that a mean loss of 9° to 12° of external rotation [9–12], and some have reported external rotation losses of up to 20° [13, 14]. Forward flexion was less consistently evaluated.

In our practice there is no significant loss of range of motion especially on external rotation probably because we protect the subscapularis tendon during surgery. An immediate postoperative rehabilitation, including external rotation, is another reason to explain these good results.

### 3.2. Satisfaction

In the literature, there are numerous reports of good results after a Bristow-Latarjet procedure. According to Rowe or Walch-Duplay scores the rates of excellent and good results go from 69% to 93% (Table 1).

### 3.3. Recurrent Instability

The Bristow-Latarjet procedure and its many modifications are relatively successful in achieving glenohumeral stability with recurrent instability reported as 0% to 5.4% (Table 1). Recurrent instability is usually defined by dislocation only.

Hill et al. [15] reported the results of 107 procedures and, at an average 58-month-follow-up, found a rate of redislocation of 3% and a rate of subluxation of 6%. Torg et al. [6] performed a modified procedure in which the coracoid process was secured to the proximal part of the glenoid rim, over the superior margin of the subscapularis. In 212 patients who had been followed for an average of 3.9 years, they found a dislocation rate of 3.8% and a subluxation rate of 4.7%

In the most recent series, Allain et al. [13] reported no recurrent dislocation but subjective subluxation in 1 of 58 shoulders (2%) at a mean follow-up of 14.3 years. Hovelius et al. [18] reported a recurrence rate of 4% for 118 shoulders (113 patients) at 15-years-follow-up and a subluxation rate of 9%.

### 3.4. Glenohumeral Arthritis

The precise etiology of osteoarthritis is unknown. It is most likely a result of initial traumatic shoulder dislocation. The risk increases with the age of the first dislocation and the number of recurrence [24].

The main factor classically associated with significant degenerative changes after the Latarjet procedure is an overhanging position of the bone block [13, 25].

### 3.5. Bone Block Position and Screw Fixation

Many authors have studied the bone block position on radiographs. Allain et al. [13] observed 53% too lateral bone blocks and 5% too medial bone blocks. Cassagnaud et al. [25] reported more than 10% of the bone blocks were found overhanging on the CT scans. Hovelius et al. [26] found 36% malpositioned bone blocks above the equator and 6% too medially placed bone blocks. Huguet et al. [27] found 45% of the grafts overhanging in the joint. All of these works showed the importance of the graft position, which is directly related to the final result.

That is, a too lateral or overhanging bone block leads to arthritis in more or less long term [6, 25–29]. A too medial bone block will result in recurrent instability [26, 27, 30], and a bone block located above the equator also exposes the joint to recurrent dislocation [26].

The optimum position is difficult to define but it is recognized that it should be below the equator, neither too medial nor too lateral: less than 10 mm from the cartilage for some [26], less than 2 mm for others [27]. For some, the bone block should really be flush to increase the articular surface of the glenoid, reduced by “crossing lesions.”

For a long time we have been using only one malleolar screw to fix the bone block and we noticed some pseudarthrosis [8] related to this screw. Since 5 years we prefer to use an AO 6.5 mm lag screw and our results are now much better than previously.

## 4. Mini-Open Technique to Arthroscopic Procedure

The open Latarjet procedure has show excellent and reliable results. The natural evolution of this procedure was to reduce the skin incision, nearly 3 to 4 centimeters, and not to cut the subscapularis tendon.

Some surgeons try to develop an arthroscopic Latarjet procedure [31, 32]. This procedure offers many advantages, including a good exposure of glenoid surface and a secure extra-articular bone block position. Moreover, if the capsule and the labrum are not resected it is possible to reattach them.

But arthroscopic Latarjet procedure, as said Boileau et al. [33], is a complex procedure that requires a steep learning curve and a certain degree of expertise and technical skill. This technique was developed by this surgeon on cadaveric specimens after 20 years of experience with the open technique.

## 5. Conclusion

Anterior stabilization of the glenohumeral joint by means of the Latarjet procedure continues to be a viable treatment option in selected patients with posttraumatic anterior shoulder instability. The results reported in the literature invariably show an easy rehabilitation, a low rate of reoperation, a good stability (a low rate of recurrent dislocation), and excellent and good subjective outcomes.

This procedure has been traditionally performed as an open technique. At the beginning, the skin incision extended to 8 centimeters and the subscapularis tendon was cut vertically. Now we limit the approach to 4 or 5 cm and when it is possible to 3 cm, for example, in thin women. This mini-open technique is not demanding for the surgeon because of the skin elasticity. In our experience the time to realize this technique do not exceed one hour. Moreover in our technique we do not cut the subscapularis tendon but we split it at its distal edge in order to place the bone block in right position. This allows a fast recovery without any postoperative immobilization.

## Figures and Tables

**Table 1:** Reported results of Bristow-latarjet procedures.

Studies	No. of patients	Follow-up (months)	Luxation rate (%)	Subluxation rate (%)	Rowe score/Walch-Duplay score			
	(No. of shoulders)				Excellent (%)	Good (%)	Fair (%)	Poor (%)
Carol et al., 1985 [16]	44 (47)	43	0	0	62	25	11	2

Banas et al., 1993 [11]	79 (79)	103	4	—	74	11	9	6
Singer et al., 1995 [14]	14 (14)	246	0	7	36	57	7	0

Pap et al., 1997 [17]	31 (31)	31	3	—	45	39	6	10

Allain et al., 1998 [13]	56 (58)	171	0	2	64	24	9	3

Hovelius et al., 2004 [18]	113 (118)	182	4	9	71	15	11	4

Matthes et al., 2007 [19]	29	38	0	3	59	24	10	7

Collin et al., 2007 [20]	74 (74)	50	5.4	2.7	18.8	49.9	20.2	10.1

Dossim et al., 2008 [21]	84 (93)	98	5.4	2.7	30	43	16	11

Edouard et al., 2010 [22]	20 (20)	21	0	0	95	0	0	5

Di Giacomo et al., 2011 [23]	26 (26)	17	0	0	69	23	8	0
